# Effects of Methylphenidate on Cognitive Function in Adults with Traumatic Brain Injury: A Meta-Analysis

**DOI:** 10.3390/brainsci9110291

**Published:** 2019-10-24

**Authors:** Yung-Jiun Chien, Yung-Chen Chien, Chien-Ting Liu, Hsin-Chi Wu, Chun-Yu Chang, Meng-Yu Wu

**Affiliations:** 1 Department of Physical Medicine and Rehabilitation, Taipei Tzu Chi Hospital, Buddhist Tzu Chi Medical Foundation, 231 New Taipei, Taiwan; jessica.kan.48@gmail.com (Y.-J.C.); ctliu242@gmail.com (C.-T.L.); hsinchiwu@gmail.com (H.-C.W.); 2 Department of Medical Education, Taipei Medical University Hospital, 110 Taipei, Taiwan; 102311050@gms.tcu.edu.tw; 3 School of Medicine, Tzu Chi University, 970 Hualien, Taiwan; 4 Department of Emergency Medicine, Taipei Tzu Chi Hospital, Buddhist Tzu Chi Medical Foundation, 231 New Taipei, Taiwan; 5 Department of Emergency Medicine, School of Medicine, Tzu Chi University, 970 Hualien, Taiwan

**Keywords:** methylphenidate, traumatic brain injury, adult, meta-analysis

## Abstract

This meta-analysis evaluated the effects of methylphenidate (MPH) on cognitive outcome and adverse events in adults with traumatic brain injuries (TBI). We searched PubMed, EMBASE, and PsycINFO for randomized controlled trials (RCTs) published before July 2019. Studies that compared the effects of MPH and placebos in adults with TBI were included. The primary outcome was cognitive function, while the secondary outcome was adverse events. Meta-regression and sensitivity analysis were conducted to evaluate heterogeneity. Seventeen RCTs were included for qualitative analysis, and ten RCTs were included for quantitative analysis. MPH significantly improved processing speed, measured by Choice Reaction Time (standardized mean difference (SMD): −0.806; 95% confidence interval (CI): −429 to −0.182, *p* = 0.011) and Digit Symbol Coding Test (SMD: −0.653; 95% CI: −1.016 to −0.289, *p* < 0.001). Meta-regression showed that the reaction time was inversely associated with the duration of MPH. MPH administration significantly increased heart rate (SMD: 0.553; 95% CI: 0.337 to 0.769, *p* < 0.001), while systolic or diastolic blood pressure did not exhibit significant differences. Therefore, MPH elicited better processing speed in adults with TBI. However, MPH use could significantly increase heart rate. A larger study is required to evaluate the effect of dosage, age, or optimal timing on treatment of adults with TBI.

## 1. Introduction

Traumatic brain injury (TBI) is one of the leading causes of mortality and morbidity in the world. It is estimated that 2 million people suffer from TBI annually in the USA, wherein it contributes to 52,000 deaths every year [1]. From 1997 to 2007, mortality rates have gradually decreased from 19.3 to 17.8 per 100,000 people in the USA [2]. Nevertheless, young and elderly adults carry a higher risk of mortality due to motor-vehicle accident and fall [2,3]. Besides, TBI survivors suffer from a wide range of neuropsychiatric sequelae including cognitive dysfunction, depression, and agitation [4]. 

Methylphenidate (MPH) is a psychostimulant that acts as a norepinephrine reuptake inhibitor and dopamine reuptake inhibitor [5]. It is most commonly used in treating attention deficit hyperactivity disorder (ADHD) and narcolepsy in children. However, the effect of MPH in treating post-TBI cognitive deficits was controversial. In 2006, a guideline recommended the use of MPH in TBI to improve attention and processing speed [6]. A recent meta-analysis including both adult and pediatric patients showed that MPH use enhanced neither memory nor processing speeds but improved attention in the treated individuals [7]. Also, pediatric TBI is associated with secondary ADHD, in which case, the effect of MPH could be obscured from the effect of treating ADHD instead of TBI [8,9]. It is, therefore, necessary to conduct a comprehensive study focusing on the effect of MPH in adults. In this meta-analysis, we aim to evaluate the effect of MPH in adult patients with TBI and provide a foundation to develop novel strategic therapies. 

## 2. Methods

### 2.1. Study Design

This is a meta-analysis of randomized control trials (RCTs) aimed at assessing the effects of MPH on cognitive functions in adults with TBI. This study complies with the recommendations made by the Preferred Reporting Items for Systematic Review and Meta-analysis (PRISMA) statement [10]. The approval of the institutional Ethical Committee was not required for the meta-analysis.

### 2.2. Search Strategy

Two authors (YJC and CYC) searched PubMed, EMBASE, and PsycINFO databases using the following key words, “Brain Injuries” or "Brain Injuries, Traumatic" or “Diffuse axonal injury” or "Craniocerebral Trauma" or "Cerebrovascular Trauma" or "Head Injuries, Closed" or "Brain Concussion" in conjunction with “Methylphenidate” or ”Methylphenidate hydrochloride” and other brand names, such as “Ritalin” or “Concerta”. The detailed search strategies are listed in supplements. The relevant studies published before July 2019 were analyzed without linguistic or geographical limitations and screened by titles, abstracts, and full texts from the electronic databases. The corresponding reference articles cited in the included studies were also used to search the additional studies. 

### 2.3. Eligibility Criteria

All studies identified from electronic databases were screened and selected by two authors (YJC and CYC) independently, as per the following inclusion criteria: (a) study should be crossover or parallel RCTs; (b) populations included more than two individuals and enrolled adult patients with TBI; (c) interventions compare MPH alone to placebo; (d) the clinical outcomes focus on cognitive function; (e) limited to human studies and no language or ethnicity restrictions were applied. Studies were excluded if they did not meet the inclusion criteria.

### 2.4. Risk of Bias in Individual Studies

Two authors (YJC and CYC) evaluated the methodological quality of all included studies independently by using the Cochrane Handbook for Systematic Reviews of Interventions. The third author (MYW) provided the consensus or discussion for disagreements. 

### 2.5. Data Extraction

The information of included studies was extracted by two authors independently (YJC and CYC), including the authors, published year, study design, number of randomized patients, patient characteristics, dose regimen of methylphenidate, cognitive outcome measurement, and adverse events. The primary outcome focused on the clinical cognitive effect of methylphenidate. The adverse events that were recorded included tachycardia, hypertension, or gastrointestinal symptoms, as secondary outcomes in our study. The detailed result is listed in Table 1.

### 2.6. Statistical Analysis

The efficacy was estimated for each study by the mean difference (MD) or standardized mean difference (SMD) for continuous data outcome. The pooled estimates with 95% CI were computed using inverse variance method with a random-effects model to account for the heterogeneity between studies. Crossover studies [11,15,16,23,26] were treated as paired groups, with the correlation coefficient between intervention and placebo set as 0.5. In studies where the outcomes were reported as the median and interquartile range [28], the sample mean and standard deviation were estimated based on previous literature [29]. Heterogeneity was assessed by Cochran Q statistic and quantified with the I^2^ statistic. Meta-analysis with high heterogeneity underwent meta-regression in continuous outcome or subgroup analysis in categorical outcome. Meta-regression using restricted maximum likelihood was performed to explore potential variables that could explain the heterogeneity. Additional sensitivity analysis using the one-study-remove approach evaluated the influence of each study on the overall effect. All the analyses were conducted using Comprehensive Meta-Analysis Version 3 [30]. *p* < 0.05 was considered statistically significant.

## 3. Results

### 3.1. Study Identification and Selection

**Figure 1:**
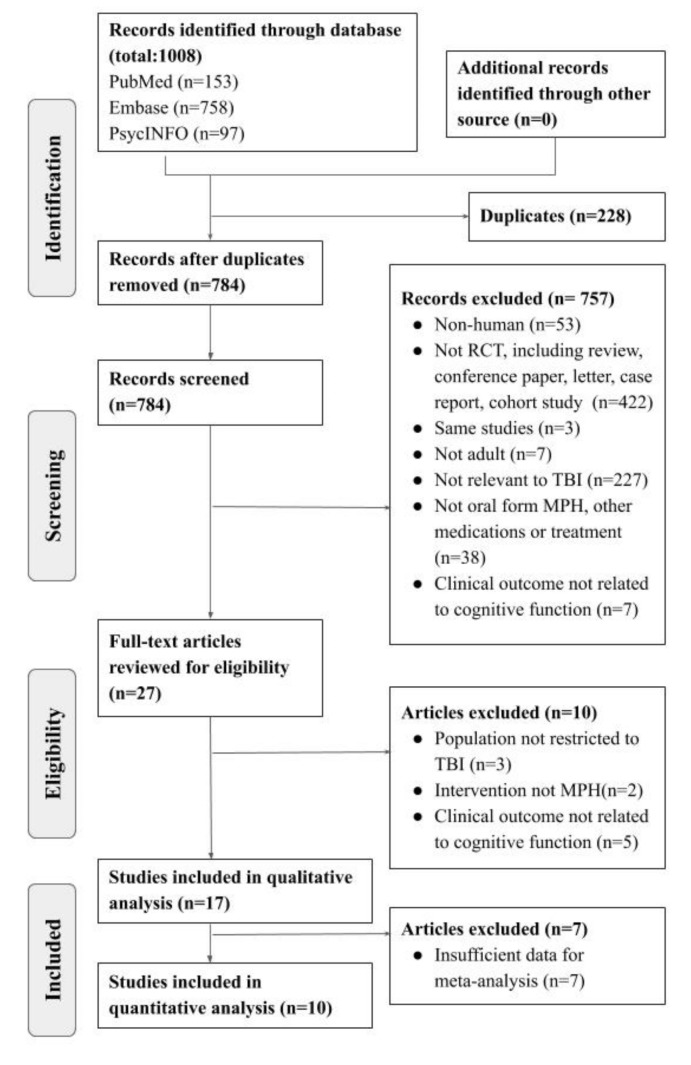
PRISMA (Preferred Reporting Items for Systematic Review and Meta-analysis) flow diagram. TBI: traumatic brain injury; MPH: methylphenidate; RCT: randomized control trials.

A total of 1008 studies were identified from major databases, including PubMed (*n* = 153), EMBASE (*n* = 758), and PsycINFO (*n* = 97). After removing 228 duplicates, the remaining studies were screened for eligibility. A total of 757 of them were excluded, owing to their lack of relevance, animal studies, or other article types. As a result, 27 studies were assessed with full-text review. A total of 17 articles were excluded due to irrelevant outcome, different populations, other article types, and the lack of exclusive methylphenidate arm. An additional seven studies were excluded due to insufficient data for meta-analysis. Finally, 10 studies involving 273 patients were used to estimate the pooled effect. The detailed PRISMA flow diagram is shown in Figure 1.

### 3.2. Study Characteristics

The characteristics of the included studies, with a total of 462 patients are summarized in Table 1. With regards to the severity of TBIs, most of the studies reported moderate to severe injuries [14,16,19,21,22,23,25,26]. Others were mild to moderate [15,18], mild to severe [11,12,24,27], and mild [17]. Two studies did not mention about the severity index [13,20]. Besides, one study was single-blinded only [20], others were randomized, double-blinded, and placebo-controlled. The regimen of methylphenidate varied across studies in terms of the dosage and frequency, from a single dose of 20–30 mg of MPH to a titrated dose for 30 weeks [27]. Measurements related to cognitive outcome also varied across studies. The adverse events including heart rate and systolic and diastolic blood pressure were assessed.

### 3.3. Quality and Risk of Bias Assessment

**Figure 2:**
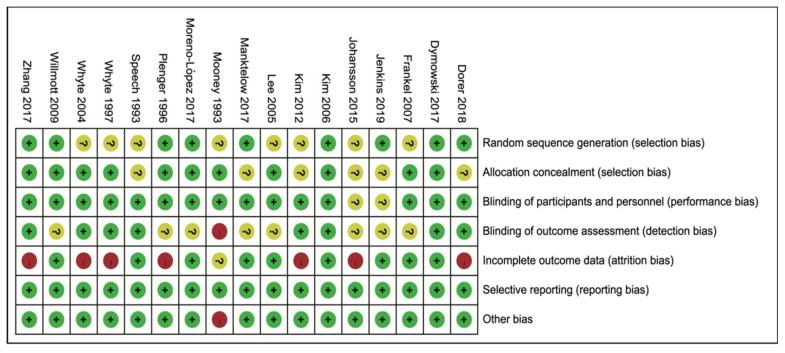
The detail risk of bias of included studies was assessed according to the Cochrane handbook.

The summary of the risk of bias in each of the included studies is listed in Figure 2. Most information is derived from the studies at low risk of bias. The work by Mooney et al. [20] is a single-blinded study, and thus involves a high risk of bias in detection bias.

### 3.4. Effects of Methylphenidate on Cognitive Function Improvement

Memory, attention, and processing speed are common long-term cognitive sequelae in patients with TBIs [31]. Various neuropsychological tests were applied to evaluate different domains of cognition in the included studies. Tests that were in more than two studies were enrolled in meta-analysis [32]. Ten tests designed to evaluate memory, attention, or processing speed were extracted from the included studies, including Choice Reaction Time, Complex Selective Reaction Time, Simple Selective Reaction Time, Trail Making Test A and B, N-back Test, Mental Arithmetic Test, Ruff 2&7 test (automatic speed raw score and controlled speed raw score), Visual Sustained Attention Task, Digit Symbol Coding Test, and Digit Span.

#### 3.4.1. Effects of Methylphenidate on Processing Speed

**Figure 3:**
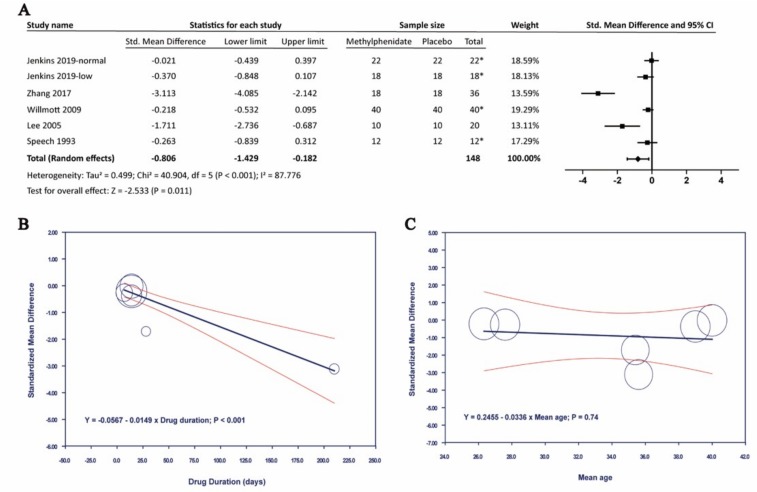
The effects of methylphenidate in Choice Reaction Time. (**A**) Forest plot of methylphenidate in Choice Reaction Time, (**B**) Meta-regression analysis of heterogeneity with drug duration, (**C**) Meta-regression analysis of heterogeneity with mean age. *: crossover study.

Five of the studies included Choice Reaction Time, which measured overall sensorimotor function and processing speed [23,26,28,33]. In Figure 3, the results showed that MPH compared with placebo has a significant effect on Choice Reaction Time, with a standardized mean difference (SMD) by random-effects model of −0.806. (95% confidence interval (CI): −1.429 to −0.182; *p* = 0.011). A high heterogeneity (I^2^ = 87.776%) was found between studies. Meta-regression with drug duration was significantly associated with improving Choice Reaction Time (*p* < 0.001). Sensitivity analysis by using one-study-remove approach did not affect the above results. 

**Figure 4:**
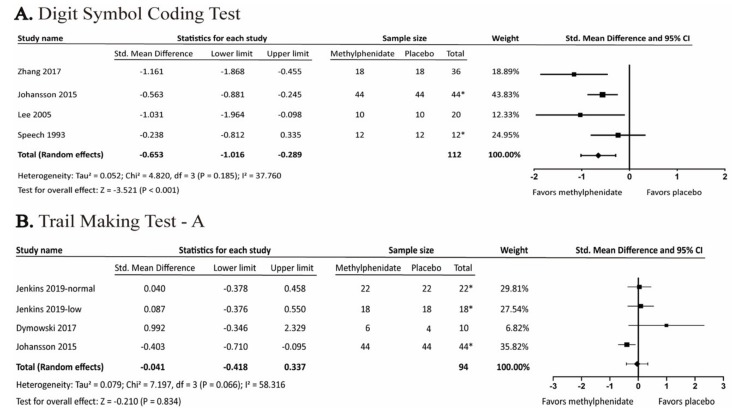
Forest plot of the effects of methylphenidate in (**A**) Digit Symbol Coding Test and (**B**) Trail Making Test, part A. *: crossover study.

Four of the studies included Digit Symbol Coding Test, also known as “Digit Symbol” [23] or “Digit Symbol Substitution Test” [18,27], which measured cognitive efficiency, visuo-motor coordination, and processing speed. Meta-analysis showed significant results with SMD of −0.653 (95% CI: −1.016 to −0.289; *p* < 0.001; I^2^ = 37.76%). We did not perform meta-regression due to the lack of a sufficient number of studies (Figure 4). On the other hand, Trail Making Test, part A, which measures processing speed [34], was not significant (Figure 4).

#### 3.4.2. Effects of Methylphenidate on Working Memory

**Figure 5:**
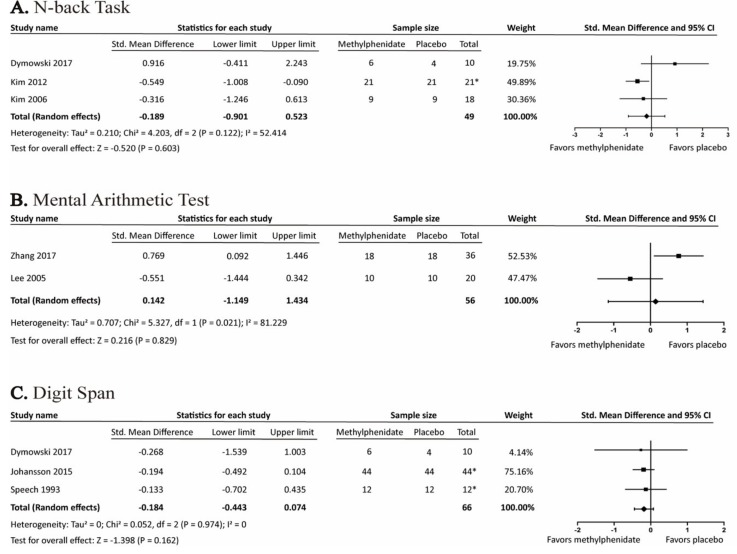
Forest plot of the effects of methylphenidate on working-memory-related cognitive tests (**A**) N-back Task (**B**) Mental Arithmetic Test (**C**) Digit Span. *: crossover study.

The effects of MPH on working memory were assessed by N-back Test, Mental Arithmetic Test, and Digit Span. None of these tests were statistically significant (Figure 5).

#### 3.4.3. Effects of Methylphenidate on Attention

**Figure 6:**
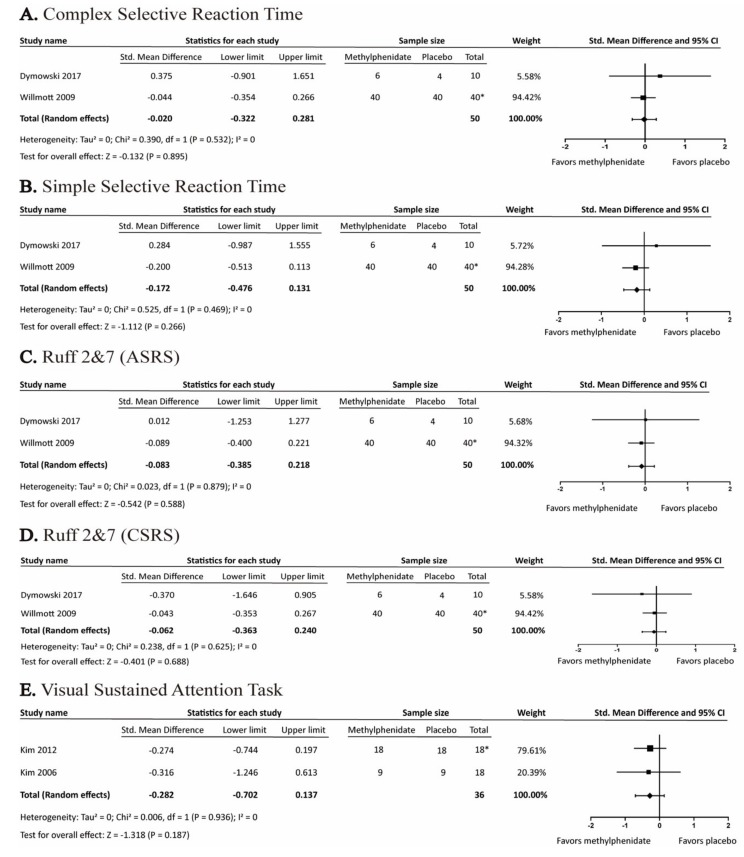
Forest plot of the effects of methylphenidate on attention-related cognitive tests. *: crossover study.

The effects of MPH on attention were assessed by Complex Selective Reaction Time, Simple Selective Reaction Time, Ruff 2&7 test (automatic speed raw score and controlled speed raw score), and Visual Sustained Attention Task. None of the tests were statistically significant (Figure 6).

### 3.5. Adverse Events of Methylphenidate in Adult Patients with Traumatic Brain Injury

**Figure 7:**
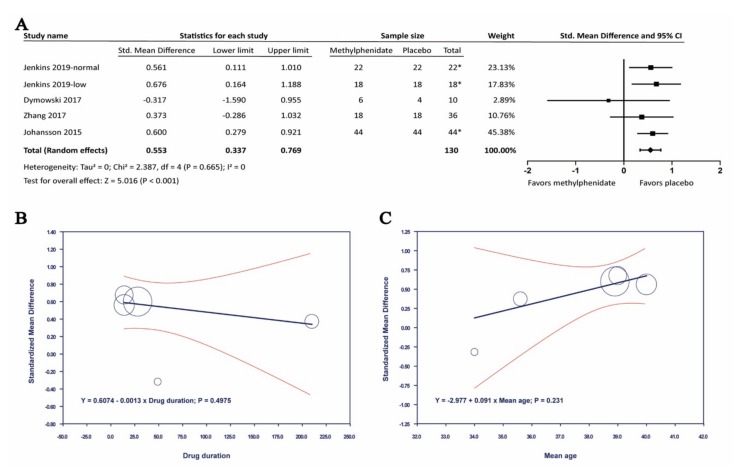
The effects of methylphenidate in heart rate. (**A**) Forrest plot of the effects of methylphenidate in heart rate, (**B**) Meta-regression analysis of heterogeneity with drug duration, (**C**) Meta-regression analysis of heterogeneity with mean age. *: crossover study.

In our included articles, the adverse effect was reported in five studies. In seven studies, there was no detail on adverse effect reported. All the recorded data was listed in Table 1. Amongst the reported studies, four articles found that tachycardia was a common adverse effect. Also, of all the reported adverse events, changes in heart rate and blood pressure were the most reported secondary outcomes in the included studies. However, none of the included studies had major cardiovascular events or life-threatening complications. Four of the studies which included heart rate as a secondary outcome showed an SMD of 0.553 (95% CI: 0.337 to 0.769; *p* < 0.001; I^2^ = 0%). Four of the studies which included heart rate as a secondary outcome showed an SMD of 0.553 (95% CI: 0.337 to 0.769; *p* < 0.001; I^2^ = 0%) (Figure 7). Meta-regression with neither drug duration nor mean age was significant. Changes in systolic blood pressure (SBP) or diastolic blood pressure (DBP) were not significant between methylphenidate and placebo groups. The headache and gastrointestinal symptoms were also reported in a few articles, including Lee 2005 [18] and Plenger 1996 [22]. But the data of other adverse symptoms were insufficient.

## 4. Discussion

### 4.1. Principle Finding

In the present study, MPH significantly improved the Choice Reaction Time, with SMD −0.806 (95% CI: −1.429 to −0.182, *p* = 0.011, I^2^ = 87.776%). Meta-regression analysis showed that the drug duration was inversely associated with Choice Reaction Time, thereby indicating an improvement in the processing speed (*p* < 0.001). MPH also benefited in Digit Symbol Coding Test, with SMD −0.653 (95% CI: −1.016 to −0.289, *p* < 0.001). Other cognitive tests in this meta-analysis were not significant. However, among the reported adverse effects, heart rate significantly increased, with SMD 0.553 (95% CI: 0.337 to 0.769, *p* < 0.001, I^2^ = 0%). On the contrary, changes in SBP and DBP were not significant upon MPH treatment.

Various tests were included for evaluating processing speed, working memory, and attention in our study. In our study, processing speed was evaluated by Choice Reaction Time and Digit Symbol Coding Test. Choice Reaction Time is commonly used due to its easy application, high test−retest reliability [35], and high prognostic value in post-TBI [36,37]. Digit Symbol Substitution Test (DSST) is a quick and reliable neuropsychological tool to evaluate cognition. Studies have shown that DSST not only relates to the severity of TBI, but also correlates well with the patient’s functional outcome [38,39]. Other tests are commonly used and well-published in previous studies to be associated with clinical outcome, however, we was unable to include them in our meta-analysis due to an insufficient number of studies, such as Glasgow Outcome Scale—extended (GOSe) and Rivermead Post Concussion Symptoms Questionnaire. The GOSe is the extended version of GOS, with eight points in total. National Institute of Neurological Disorders and Stroke had recommended the use of GOSe as an outcome measurement after TBI [40]. Rivermead Post Concussion Symptoms Questionnaire is used to evaluate post-concussion symptoms, which include cognition, sleeping quality, mood, and other physical symptoms [41]. However, GOSe and Rivermead Post Concussion Symptoms Questionnaire were only included in one study respectively [14,18]. More RCTs will be needed to perform further analysis with these two measurement parameters.

### 4.2. Comparison with Other Studies

Our findings of Choice Reaction Time and Digit Symbol Coding Test were compatible with the guidelines of 2006, which recommended methylphenidate use in TBI for improving attention and processing speed [6]. Previous meta-analysis that involved both children and adult patients showed no benefits in memory or processing speed. 

However, methylphenidate had been used in treating ADHD, a common sequalae after pediatric TBI [42,43]. It is reported that 19%-48% of pediatric patients who had suffered from TBI developed secondary ADHD [44]. Also, adult and pediatric brains could be very different. Children’s brains have higher degrees of neuroplasticity and change rapidly during development [45]. Therefore, pediatric patients may have different outcome or recovery compared with adults. In our study, we clarified the beneficial effect of MPH in processing speed in adults with TBI. 

Moreover, MPH was significantly associated with cognitive improvement over time, measured by Choice Reaction Time in our study. The effect of long-term MPH use in patients with TBI was not clear. Our study supported a previous RCT that reported cognitive improvement in adult patients with TBI after long-term treatment of MPH [46]. But, an animal study had found that chronic use of MPH was associated with increasing oxidative stress and neuroinflammation in brain [47]. Current studies deciphering long-term MPH effects on cognition outcome are controversial and limited. 

Besides, changes in the heart rate were significantly associated with MPH, even though it is not associated with any major cardiovascular events in our study. Cardiovascular adverse effects of MPH had been a concern since it was first reported in 1958 [48]. MPH had a sympathomimetic property which could activate beta-adrenoreceptor on cardiac tissues [49]. However, elevated heart rate is a risk for major cardiovascular disease, and all of which could lead to death [50]. Our findings supported a recent meta-analysis which suggested close monitoring of heart rate and SBP throughout the treatment with MPH in ADHD [51]. 

### 4.3. Mechanism of TBI-Related Cognitive Deficits and MPH Effect

TBI-related cognitive deficits were determined by the extent of damage from direct and indirect injuries or primary and secondary injuries. Direct injuries or primary injuries occurred during initial physical impact, causing irreversible damage. Diffuse lesions such as diffuse axonal injury were caused by acute rotational acceleration and deceleration [52]. Focal lesions were mostly located in frontal and temporal regions, where it is adjacent to the bony structure of petrous ridges and prominence [31]. Damaging the frontal lobe and temporal lobe could impair attention, executive function, and memories, which were some of the most disturbed symptoms after TBI. Indirect injuries or secondary injuries occurred hours to weeks after the initial physical impact. Secondary injuries also played a role in poorer outcome [53] by initiating complex cascades of glutamate excitotoxicity, excessive calcium influx, neuroinflammation, and pro-apoptosis pathway [54].

On the other hand, dysfunction of the neuromodulator system including dopamine and noradrenaline may lead to persistent cognitive deficits after TBI [55]. MPH increased the extracellular concentration of dopamine and norepinephrine in pre-frontal cortex by blocking dopamine transporter and norepinephrine transporters [56]. However, preclinical studies demonstrated an inverted-U dose−response relationship between prefrontal dopamine activity and working memory [57]. Stimulants at low dose increased dopamine level and enhanced arousal, attention, and improved cognition; while high doses could lead to cognitive impairment. Jenkins et al. further demonstrated the effect of MPH in patients with a hypodopaminergic state comparing to a normo-dopaminergic state [28]. Since most of the studies did not evaluate the dopamine state in patients with TBI, controversial clinical results of MPH effect were inevitable.

## 5. Strengths and Limitations

The major strength in our study was that only RCTs which enrolled adult patients were included. Age-dependent effects of MPH had been studied in pre-clinical studies [58] as well as recent MRI studies [59]. Also, brain in children or adolescents is still undergoing development and greatly differed with respect to brain plasticity and was influenced by environmental factors [60,61]. We chose to select RCTs with adult patients to limit the differences in age and neurobiological system [61]. On the other hand, we enrolled RCTs with careful evaluation of risk of bias to minimize the bias in this study. 

Besides the strength, our study had several limitations. First, the heterogeneity of the included studies limited the significance of the study. The included RCTs had different MPH dosage, follow up time, and disease severity, which may influence the final results. Outcomes of our study may be underestimated or overestimated. Secondly, due to a limited amount of RCTs, funnel plot was not performed. While we have carefully evaluated the risk of bias in included RCTs, undetected publication bias may be still present. Thirdly, some of the cross-over studies did not provide enough wash-out period. MPH is metabolized in liver and excreted mostly through urine after nearly 48–96 h [62]. The study without enough wash-out periods could hardly exclude carryover effects. Again, the results of the meta-regression should be interpreted carefully. We conducted meta-regression to explore the heterogeneity with the continuous data reported in each outcome. However, lack of a sufficient amount of studies could lead to type 1 error. Above all, large scales of RCTs are warranted for further research.

## 6. Conclusions

This meta-analysis showed that MPH had a significant effect in improving processing speed in adults with TBI, especially with longer drug duration. Other tests that involved working memory and attention were not significant. Although MPH use could significantly increase the heart rate, no major cardiovascular events were reported. We concluded that MPH should be administered in adult patients with TBIs with regularly monitoring heart rate. RCTs with a larger sample size will be needed to support our findings and explore the potential effects of MPH on other domains of cognitive function. 

## Figures and Tables

**Table 1:** The detailed characteristics of included studies.

Study	Severity	Age	Size	Study Design	Dose Regimen	Measurements Related to Cognitive Outcome	Adverse Events
Dorer 2018 [11]	Mild to severe TBI for more than 6 months	19–58	28	Double-blind, placebo-controlled, crossover study	30 mg, one dose	Rapid Visual Information Processing Task N-back test Stop Signal Tower of London	No available data
Dymowski 2017 [12]	Mild to severe TBI at least 6 months	16–65	11	Randomized, placebo-controlled, double-blind trial	0.6 mg/kg QD extended-release methylphenidate for 7 weeks	National Adult Reading Test Symbol Digit Modalities Test N-back test Trail Making Test Digit Span Hayling Test Ruff 2 and 7 Selective Attention Test Computerized Selective Attention Task	Trend to increase BP and anxiety
Frankel 2007 [13]	Severity not mentioned for 9 years and 10 years respectively	40 and 49	2	Randomized, placebo-controlled, double-blind trial	25 mg QD for 2 weeks	The Stroop Color Word Test Echopraxia Tasks The Face Recognition Task The Self Ordered Pointing Test Verbal Paired Associates Word Lists The Neuropsychology Behavior and Affect Profile Controlled Oral Word Association Test Category Naming	No available data
Jenkins 2019 [14]	Moderate to severe TBI for at least 3 months	20–65	40	Randomized, double-blind, placebo-controlled, crossover study	0.3 mg/kg BID for 2 weeks	Choice reaction time Trail Making Test Delis-Kaplan Executive Function System The Stroop Color-Word Interference Test The People Test The Wechsler Abbreviated Scale for Intelligence Matrix Reasoning and Test for Adult Reading Lille Apathy Rating Scale Visual Analogue Scale for Fatigue Glasgow Outcome scale-extended Hospital Anxiety and Depression Scale Frontal Systems Behavioral Scale.	Restlessness, increased heart rate
Johansson 2015 [15]	40 mild TBI and 4 moderate TBI for more than 6 months	18–65	44	Randomized, crossover study	No medication 4 weeks, low dose (5 mg TID) 4 weeks, normal dose (20 mg TID) 4 weeks.	Mental Fatigue Scale Visual Analogue Scale for Pain Comprehensive Psychopathological Rating Scale Digit symbol Coding Test Digit Span Trail Making Test Short Form-36	Increased BP, restlessness, depressive symptoms. No serious events.
Kim 2012 [16]	Moderate to severe TBI for at least 3 months	16–60	23	Randomized, double-blind, placebo-controlled crossover study	0.3 mg/kg one dose	Visual sustained attention task Two-back task	No available data
Kim 2006 [17]	Mild TBI for at least 6 months	16–60	18	Randomized, double-blind, placebo-controlled trial	20 mg one dose	Two-back task Visuospatial attention task	No patient complained about uncomfortable side effect
Lee 2005 [18]	Mild to moderate TBI for at least 2 months but no longer than 1 year	18–55	30	Randomized, double-blind, placebo-controlled trial	Methylphenidate starts at 5 mg/day to 20 mg/day in a week / sertraline starts 25 mg /day and increased to 100 mg/day in a week / placebo for 4 weeks.	Beck Depression Inventory Hamilton Depression Rating Scale Rivermead Postconcussion Symptoms Questionnaire SmithKline Beecham Quality of Life Critical Flicker Fusion Choice Reaction Time Continuous Tracking, Mental Arithmetic Short-Term memory Digit Symbol Substitution Test Mini-Mental State Examination Leeds Sleep Evaluation Questionnaire Epworth Sleepiness Scale	Nausea/vomiting, diarrhea, constipation, palpitation, sweating
Manktelow 2017 [19]	Moderate to severe TBI for at least 6 months	18–60	30	Randomized, double-blinded, placebo-controlled, crossover study	Single dose of 30 mg	Spatial Span Paired Associates Learning Intra/Extradimensional Set Shift Simple Reaction Time	No available data
Mooney 1993 [20]	Severity not mentioned at least 6 months	18–50	38	Randomized, placebo-controlled group, single-blind trial	Gradually added to 30 mg per day for 6 weeks	State-Trait Anger Scale The Belligerence cluster score from the Katz Adjustment Scale (KAS-Belligerence) The Anger-Hostility factor score of the Profile of Mood States (POMS-Anger Hostility) Letter Cancellation test Selective Reminding Test The General Psychopathology cluster score of the Katz Adjustment Scale (KAS-General Psychopathology) The Organic Signs and Symptoms Inventory (OSSI) The Recent Experience Checklist	No difference evaluated by The Recent Experience Checklist
Moreno-López 2017 [21]	Moderate to severe TBI for at least 7 months	36.86 in average	34	Randomized, double-blinded, crossover study	30 mg single dose	Spatial Span Test Intra-extra Dimensional Set Shift Stop-signal Task	No available data
Plenger 1996 [22]	Moderate to severe TBI or complicated mild TBI, subacute stage	16–64	23	Randomized, double-blind, placebo-controlled trial	0. 3 mg/kg BID for 30 days	Disability Rating Scale Galveston Orientation and Amnesia Test Continuous Performance Test 2 & 7 Test Paced Auditory Serial Addition Test Digit Span & Attention/Concentration from Wechsler Memory Scale-Revised (WMS-R) Selective Reminding Delayed, Verbal and Visual Memory from the WMS-R Proteus Maze Pursuit Rotor Symptom Interview	insomnia, headache
Speech 1993 [23]	Moderate to severe TBI for 73 to 102 months	> 12	12	Randomized, double-blind, placebo-controlled crossover study	0.3 mg/kg BID for 1 week, then cross-over	Gordon Diagnostic System Digit Symbol Digit Span Stroop Interference Task Two-choice complex reaction time task The Sternberg High Speed Scanning Task Selective Reminding Test Serial Digit Test Katz Adjustment Scale	No patients report side effect
Whyte 1997 [24]	Mild to severe TBI for 38 to 3245 days	17–75	19	Randomized, double-blind, placebo-controlled trial	0.25 mg/kg BID for 2 days	The Sustained Arousal Task The Phasic Arousal Task The Distraction Task The Choice Reaction Time Task Behavioral Inattention.	No available data
Whyte 2004 [25]	Moderate to severe TBI for at least 3 months	16–60	34	Randomized, double-blind, placebo-controlled, crossover study	0.3 mg/kg BID for 6 weeks	Sustained Arousal and Attention Task Speed/Accuracy Tradeoff Task Distraction Task Choice Reaction Time Task Dual Task Sustained Attention to Response Task Test of Everyday Attention Inattentive Behavior Task	No available data
Wilmott 2009 [26]	Moderate to severe TBI for averaged 68 days	16–60	40	Randomized, double-blind, placebo-controlled, crossover study	0.3 mg/kg BID for 2 weeks	Ruff 2 and 7 Selective Attention Test Selective Attention Task Letter Number Sequencing Task Symbol Digit Modalities Test Four Choice Reaction Time Task Sustained Attention to Response Task Wechsler Test of Adult Reading Rating Scale of Attentional Behavior Side Effects Questionnaire	Evaluated by Side Effects Questionnaire (The safety data was published in separate studies)
Zhang 2017 [27]	Mild to severe TBI for 2 weeks to 1 year	18–65	36	Randomized, double-blinded, placebo-controlled trial	Starting from 5 mg/day and gradually titrated to 20 mg/day for 30 weeks	Mental Fatigue Scale Choice Reaction Time Compensatory Tracking Task Mental Arithmetic Test Digit Symbol Substitution Test Mini-Mental State Examination Beck Depression Inventory Hamilton Rating Scale for Depression.	No difference in heart rate, BP, body weight between groups

Abbreviations: TBI = traumatic brain injury, QD = once per day, BID = twice per day, TID = three times per day
